# Is Pupil Activity Associated With the Strength of Memory Signal for Words in a Continuous Recognition Memory Paradigm?

**DOI:** 10.3389/fpsyg.2021.686183

**Published:** 2021-11-23

**Authors:** Jorge Oliveira, Marta Fernandes, Pedro J. Rosa, Pedro Gamito

**Affiliations:** ^1^Digital Human-Environment Interaction Lab, Lusófona University, Lisbon, Portugal; ^2^Psychology Unit, Universidade da Madeira, Funchal, Portugal

**Keywords:** pupillary response, recognition memory, memory strength, eye tracking, pupillometry

## Abstract

Research on pupillometry provides an increasing evidence for associations between pupil activity and memory processing. The most consistent finding is related to an increase in pupil size for old items compared with novel items, suggesting that pupil activity is associated with the strength of memory signal. However, the time course of these changes is not completely known, specifically, when items are presented in a running recognition task maximizing interference by requiring the recognition of the most recent items from a sequence of old/new items. The sample comprised 42 healthy participants who performed a visual word recognition task under varying conditions of retention interval. Recognition responses were evaluated using behavioral variables for discrimination accuracy, reaction time, and confidence in recognition decisions. Pupil activity was recorded continuously during the entire experiment. The results suggest a decrease in recognition performance with increasing study-test retention interval. Pupil size decreased across retention intervals, while pupil old/new effects were found only for words recognized at the shortest retention interval. Pupillary responses consisted of a pronounced early pupil constriction at retrieval under longer study-test lags corresponding to weaker memory signals. However, the pupil size was also sensitive to the subjective feeling of familiarity as shown by pupil dilation to false alarms (new items judged as old). These results suggest that the pupil size is related not only to the strength of memory signal but also to subjective familiarity decisions in a continuous recognition memory paradigm.

## Introduction

Pupillometry has long been used in cognitive science as a measure of cognitive activity (Sirois and Brisson, 2014). This relationship was established in the 1960s, with evidence for associations between pupillary response and psychological processes such as arousal (Hess and Polt, 1960) and short-term memory (Kahneman et al., 1968). This interest has increased rapidly ever since, mainly not only due to its recording simplicity and non-intrusiveness compared with electrophysiological measurements but also due to the automaticity of pupillary response, which is associated with autonomous nervous system activity (Steinhauer et al., 2004) being controlled in the brain by the superior colliculus (Wang and Munoz, 2015) and the locus coeruleus norepinephrine system (Joshi et al., 2016; Lewandowska et al., 2019).

The increasing interest on the relationship between pupil activity and memory processing is found in more recent debates (Brocher and Graf, 2017; Kafkas and Montaldi, 2017), which is revealed by a pupil dilation effect to familiar stimuli compared with unfamiliar stimuli.

In recognition memory designs, stimuli are encoded in a learning or study phase, being subsequently recognized in a test phase, where the (old) stimuli are intermingled with (new) interference stimuli. Studies using pupillary activity as an index for memory typically found an increase in pupil size for correctly recognized “old” stimuli relative to correct rejections of “new” stimuli in study-test procedures (Heaver and Hutton, 2011; Kafkas and Montaldi, 2012). This is known as the pupil old/new effect (Võ et al., 2008; for a review, van der Wel and van Steenbergen, 2018), which is considered as an outcome of the strength of memory signal associated with the retrieval of declarative memory (Papesh et al., 2012).

Otero et al. (2011) aimed at understanding the cognitive processes underlying pupil old/new effects in recognition memory by conducting various experiments manipulating the strength of memory signal for deep vs. shallow encoded items. The results revealed that the pupil old/new effect was more pronounced for remembered words (deeper encoding) compared with known words (shallow encoding). Brocher and Graf (2016) also demonstrated pupil old/new effects irrespective of lexicality, word valence, and frequency. More importantly, weakening the memory trace across these experiments, either by repeating legal vs. pseudowords or asking participants to make speeded responses, led to a reduction in pupil old/new effects, suggesting that conditions weakening memory signal would affect pupillary response.

Kucewicz et al. (2018) measured pupil size during encoding and recall of word lists. The lists consisted of 12-word items that were sequentially presented on a computer screen in the study phase. A distractor task was included between the study and test phases for interference. In the test phase, the participants were asked to verbally recall the word lists as fast as possible within 30 s. The authors studied the time course of pupillary response throughout the experimental task to examine the pupil dynamics for successfully recalled items compared with forgotten items. At the encoding phase, the results revealed an initial constriction followed by a pupil dilation, which increased as the word items were actively retained in memory. Moreover, an increase in pupillary response was found during word recall with the following decrease in pupil size as word items were being recalled, described as being related to the retrieval of information from memory.

Magliero (1983) and van Rijn et al. (2012) have conducted pupillometry studies manipulating the retention interval to evaluate the association with memory strength, where they found that longer retention levels increased task-evoked pupil responses. van Rijn et al. (2012) repeated the presentation of word lists with retrieval cues of paired associates in four repetitions of test trials to study the effects of repetition on the pupillary response. The results were intriguing, suggesting that repetition of word lists decreased pupillary response at retrieval. The differences between short and long retention intervals decreased with the repetition of word lists. The overall results suggest an association with retrieval effort given the effects of retention interval and repetition of word lists, supporting the hypothesis that the magnitude of pupil dilation is associated with memory strength for individual items, but in a reversed pattern than the one observed in pupil old/new effect studies.

To further explore the pupil old/new effects, Kafkas and Montaldi (2015) found that pupil activity distinguished between objective (i.e., veridical old/new status of the item) and subjective (i.e., subjective old/new decision) familiarity and novelty in two distinct temporal components. One early component was found for the objective status, while a late component near the recognition response was found for the subjective status of items, which indicates that pupil activity may be sensitive to both explicit and implicit components of recognition memory.

This study evaluates the relationship between pupil activity and recognition memory in a running recognition task (Shepard and Teghtsoonian, 1961) with varying retention intervals to assess pupil activity during explicit manipulations of memory strength. In such a task, participants should retain information that is presented in a continuous sequence of items until the test trial for memory retrieval. This task may provide a more ecological way to assess human memory processing while maximizing interference compared with recognition memory of word lists where the study-test phases are separated by isolated interference tasks. This paradigm was used earlier in behavioral studies to manipulate the retention interval in visual word recognition (e.g., Shepard and Teghtsoonian, 1961; Coney and MacDonald, 1988; Federmeier and Benjamin, 2005), but this is the first study to use the continuous recognition memory paradigm in pupil research. According to the strength account, we would expect the recognition performance and pupil dilation to decrease as the retention interval increases. Our intent is also to explore the pupil dynamics in a continuous recognition memory design by assessing pupillary responses to the objective and subjective old/new status of word items.

## Materials and Methods

### Participants

The sample comprised 42 adult Portuguese native speakers who had normal vision or corrected-to-normal vision, mostly women (*n* = 23) with a mean age of 26 years (*SD* = 6.79) and no less than 12 years of formal education. The participants were selected in a university campus for voluntary participation in a study related to “visual perception and memory.” The exclusion criterion was history of psychiatric disorder or medication/drug use. The initial pool comprised 47 participants, but five participants were excluded due to low quality (more than 50% of data loss) of pupillary recordings or due to problems in the collection of behavioral responses.

### Materials and Design

The stimulus words were collected from a database of validated Portuguese words from a sample of undergraduate students (Marques et al., 2007). For this study, we selected 107 words of 4 to 7 letters in length: 64 of these were used as study words and 43 as “new” test words. Both lists of words were matched for psycholinguistic variables of familiarity and age of acquisition.

### Design and Procedure

This study was approved by the ethics committee of the host institution where it was carried out. The experiment was conducted in a soundproof booth with a constant low-bright room during only one session. The visual word recognition task was based on a continuous recognition memory paradigm originally from Shepard and Teghtsoonian (1961), with study words presented two times in a study-test procedure. In our task, study words were repeated in the test phase, intermingled with (new) interference words with different retention intervals. All participants were tested with words presented at four different interval levels manipulated through the number of words between study and test: lag 1 (immediate repetition), lag 4 (4 words separating study-test phases), lag 8 (8 words), and lag 32 (longest lag with 32 words between the study-test phases).

Each trial in the study phase began with a fixation cross for 250 ms preceding the word stimulus that was on the screen for 1,750 ms. In the test phase, each trial began with a mask consisting of a row of seven symbols (“&&&&&&&”) for 250 ms, being replaced by the word stimulus (1,750 ms), according to the design of Heaver and Hutton (2011). All stimuli were presented at the center of the screen. The word stimulus in the test phase was followed by the mask that remained on the screen until a response was given. The recognition responses were given at this stage. The participants were instructed to respond with the keypress only when the word stimulus was replaced by the mask and during the time, the mask was visible on the screen. Following each word in the test phase, the participants also had to indicate their level of confidence in the decision (1, not at all confident to 5, very confident). Each trial of the study phase consisted of the mask and the word stimulus, whereas in the test phase, word stimuli were replaced by the mask (where recognition response was given) followed by the confidence level screen. The interstimulus interval was 1,000 ms for both the study and test phases. This procedure was the same between the different retention intervals. The only difference between retention conditions was the number of intervening items between the study and test phases. Intervening items were the number of words in a continuous sequence that comprised study words and “old” and “new” test or interference words. An example of the continuous recognition memory procedure is shown in the following sequence, where each letter describes a different word and the question mark the test phase:


a⁢b⁢c⁢a⁢?⁢c⁢?

**FIGURE 1:**
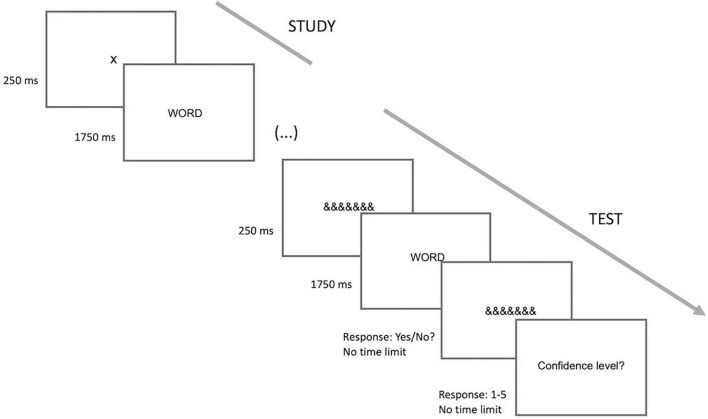
Design of the experimental task.

In this sequence, “a” is tested at a lag of 3 and “c” is tested at a lag of 2 words between the study and test phases. This design is also illustrated in Figure 1.

The words were presented in black capital letters (38-point Arial font) over a gray background screen (Red = 128, Blue = 128, and Green = 128).

After informed consent, each participant was seated at a distance of 60 cm from the infrared eye-tracking system (Tobii T60, Tobii Technology AB, Danderyd, Stockholm, Sweden; instrument noise, 0.06 RMS). The calibration of the eye tracker was carried out for each participant using a five-point calibration setup.

Participants were instructed to keep still to minimize data loss due to head and body movements during the task. Following this stage, the participants completed a 5-min preliminary practice stage using proper nouns as stimuli before the recognition task. They were instructed to indicate in the keyboard whether a word was old (previously seen during the experiment) or not, as fast as possible.

The visual word recognition task was designed in Superlab (version 1.0.2; Cedrus Corporation, San Pedro, CA, United States) and presented through the 17-inch monitor of the eye tracker with a 1,280 × 1,024 resolution. The behavioral measures were collected using Superlab, and pupil responses were registered in Tobii Studio (version 3.0; Tobii Technology AB, Sweden), which is the native application of Tobii eye trackers. Eye data of both eyes were collected at a sampling rate of 60 Hz.

### Data Pre-processing

Raw pupil data were exported from Tobii Studio version 3.3.2 software to SPSS (Version 25.0. Armonk, NY: IBM Corp.) for data reduction. The proportion of the missing values was first analyzed to assess the noise in pupil data (missing data = 3.97%). Missing pupil data were randomly distributed across trials. Pupil amplitude artifacts (<1 or >9 mm), as well as drifts and blinks, were coded as missing values (Rosa et al., 2015). Pupil diameters of zero lasting between 100 and 600 ms were considered blinks (Cosme et al., 2021), and replaced using linear interpolation (Carvalho and Rosa, 2020). Finally, a seven-point weighted average filter was applied to smooth data. The data file was then exported to Vision Analyzer software (version 2.1; Brain Products GmbH, Germany) for data segmentation and estimation of evoked pupil responses. The epochs were created for each stimulus category with stimulus-locked segments of 4,000 ms in length (i.e., from −250 to 3,750 ms at stimulus onset). This segmentation resulted in 64 segments, 16 segments for the study words tested at each of the four retention levels, plus 43 segments for the “new” test words (interference words were presented only during the test phase), in a total of 107 segments. The words at retrieval were visible during the first 1,750 ms of this time window. The remaining interval between 1,750 and 3,750 ms comprised the recognition response.

Pupil responses were calculated within each time bin of 250 ms for a time window of 3,750 ms. The baseline was set at −250 ms before the stimulus onset. The percentage of variation relative to baseline was calculated to depict the amplitude of pupillary responses to each experimental condition.

The behavioral measures consisted of accuracy from the signal detection theory (SDT), which comprises hits, correct rejections, false alarms, and misses. According to the SDT, hits and correct rejections depict correct decisions, whereas false alarms and misses are incorrect decisions that may be due to internal/external factors affecting human perception. Reaction times and confidence ratings were also assessed during this task.

## Results

### Behavioral Measures

The analysis on behavioral measures was conducted for discrimination ability, reaction times, and confidence ratings in recognition responses. These variables were analyzed by retention intervals using repeated-measures ANOVA. Confidence levels were also assessed with receiver-operating characteristics (ROC) for determining the ability to distinguish recognition responses.

### Recognition Accuracy

Recognition accuracy was calculated according to the SDT through *d*-prime (*d’*) in which higher values describe better memory performance, which is given by the following expression: *d’* Z(H)-Z(FA). Participants had an average hit rate (correct recognition) of 81% (ranging from 37 to 100%) and a false alarm rate of 11% (ranging from 0 to 29%).

The effect of retention interval on recognition accuracy was analyzed with a single-factor repeated measures ANOVA with four levels (retention level: 1, 4, 8, and 32 items). The ANOVA showed significant differences with Greenhouse-Geisser correction in recognition accuracy between retention levels [*F* (1.430, 58.611) = 16.947; *p* < 0.001; η*^2^_*p*_* = 0.292], suggesting a significant decrease (Bonferroni corrected pairwise comparisons) from lag 1 to lag 4 (*p* = 0.020) and from lag 4 to lag 8 (*p* = 0.002). Table 1 describes recognition performance in the running recognition task through *d*-prime, hits, false alarms, confidence levels, and reaction times across lag conditions. Table 2 depicts the inference statistics for these analyses. The same pattern of results was observed for hits [*F* (2.551, 107.159) = 10.591; *p* < 0.001; η*^2^_*p*_* = 0.201] and false alarms [*F* (2.740, 115.86) = 3.698; *p* < 0.05; η*^2^_*p*_* = 0.081].

**TABLE 1:** Descriptive statistics for behavioral measures.

	Lag 1		Lag 4		Lag 8		Lag 32	
	*M*	*SE*	*M*	*SE*	*M*	*SE*	*M*	*SE*
d’	3.19	0.19	2.35	0.20	1.98	0.18	1.97	0.16
Hits	0.87	0.03	0.82	0.03	0.81	0.03	0.72	0.03
FA	0.14	0.02	0.15	0.03	0.20	0.03	0.19	0.02
CR	4.78	0.48	4.56	0.67	4.56	0.62	4.21	0.09
RT	1305.84	130.78	1613.07	142.80	1619.71	139.15	1612.46	147.53

*M, mean; SE, standard error for the mean; d’, d-prime for accuracy; CR, confidence ratings; RT, reaction times; and FA, false alarms.*

**TABLE 2:** Inference statistics for behavioral measures.

	*MSE*	η*^2^_*p*_*	*F* ^a^	Pairwise^b^
d’	29.007	0.292	16.947***	l1 > l4 > l8,l32
Hits	0.190	0.201	10.591***	l1,l4 > l8,l32
FA	0.039	0.081	3.698*	l1,l4 < l8,l32
CR	3.190	0.397	27.006***	l1 > l4,l8 > l32
RT	1473240.24	0.143	6.836**	l1 < l4,l8,l32

*MSE, mean square error; η^2^_p_, effect size through partial eta squared; and F, analysis of variance statistic; l1, lag 1; l4, lag 4; l8, lag 8; l32, lag 32.*

*^a^Greenhouse-Geisser correction. ^b^Bonferroni corrected pairwise comparisons.*

*p < 0.05;

**p < 0.01;

***p < 0.001.

### Confidence Ratings

The confidence levels in each of the recognition decisions were rated on a five-point Likert scale. The same design was used for the ANOVA that showed a similar pattern to that of the *d*-prime. These results indicated a decrease in confidence level for longer retention levels [*F* (2.168, 88.905) = 27.006; *p* < 0.001; η*^2^_*p*_* = 0.397]. Pairwise comparisons with Bonferroni correction indicated that the confidence level was highest in lag 1 and lowest in lag 32. Confidence level decreased from lag 1 to lag 4 (*p* = 0.001) and from lag 8 to lag 32 (*p* < 0.001).

A descriptive analysis on confidence ratings showed that most responses were extreme-confident responses. This pattern has limited further analyses between pupil data and confidence ratings, given the lack of valid cases in each cell for factorial designs. We have conducted a ROC analysis on confidence ratings to understand whether confidence would discriminate successful recognition. This analysis showed a poor discriminant ability of confidence ratings on the recognition ability (AUC = 0.546; *SE* = 0.040; *p* = 0.249).

### Reaction Time

Reaction time was also assessed through the same ANOVA to test the significant differences between lag conditions. The ANOVA revealed a significant difference in reaction times across retention levels [*F* (2.056, 84.298) = 6.836; *p* = 0.002; η*^2^_*p*_* = 0.143], with faster responses for words tested immediately at lag 1 that differed from the remaining conditions (all *p*’s < 0.05).

### Pupillometry

Pupil size analysis was performed in different steps. First, the analysis was conducted for pupillary responses to each lag condition. Second, the pupil old/new effect was calculated by comparing correct recognition responses to “old” words with correct rejections of “new” test words. Following these analyses, the pupillary responses were analyzed for recognition errors, namely, false alarms, i.e., incorrect rejections of new test words and misses, i.e., omissions in recognizing old words. The factor related to confidence levels in recognition was not included in the factorial design due to the insufficient number of trials for low confidence conditions, but this factor was controlled in further analyses by dividing the five-point Likert scale in a dichotomous variable for low and high confident decisions. Therefore, pupillary responses to false alarms were analyzed by confidence (low vs. high) to study whether the pupil activity is also associated with subjective familiarity (i.e., evaluating “new” test items as “old”). Finally, the pupillary responses across lag conditions were also studied for extreme-confident decisions (i.e., confidence rating equal to 5).

### Pupil Dynamics by Retention Interval

Evoked pupillary responses for correct recognition decisions were analyzed to each retention condition (study-test lag) by plotting peak activity at 250 ms bins of the 3,750 ms time windows with a two-factor ANOVA. The retention level (4 levels) and bin (16 levels) were entered in this analysis as factors within-subjects.

**FIGURE 2:**
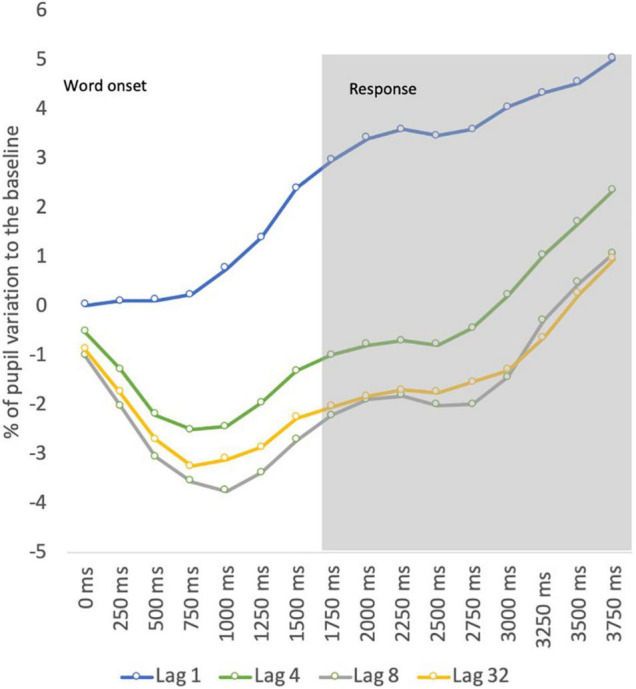
Pupil dynamics by retention interval.

The ANOVA revealed significant main effects for lag [*F* (1.718, 189.211) = 33.896; *p* < 0.001; η*^2^_*p*_* = 0.453] and bin [*F* (2.776, 189.211) = 23.939; *p* < 0.001; η*^2^_*p*_* = 0.369]. The main effect of lag described a decrease in pupil dilation for longer retention spans, whereas the main effect of bin described a pupil constriction at the initial stage of memory retrieval followed by a later dilation. This analysis also showed a significant interaction effect between factors [*F* (4.605, 189.211) = 5.949; *p* < 0.001; η*^2^_*p*_* = 0.127], suggesting a different pattern of pupil dynamics according to the retention condition. Pairwise comparisons (Bonferroni corrected) for retention level suggested a stronger pupil constriction for lags (all *p*’s < 0.05) other than lag 1, and a later dilation for all retention conditions (all *p*’s < 0.05). The differences were found mostly between lag 1 and the remaining lag conditions. This pattern is illustrated in Figure 2.

### Pupil Old/New Effect

**FIGURE 3:**
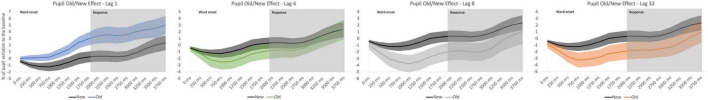
Pupil old/new effect.

To further explore these results, the differences between evoked pupillary responses to “old” test words and “new” test words were calculated for studying the pupil old/new effect observed previously in recognition memory studies. The pupillary responses to each retention condition were compared with interference test words through a separate repeated measures ANOVA. The ANOVAs revealed the pupil old/new effect only at lag 1 [*F* (2.948, 120.883) = 6.972; *p* < 0.001; η*^2^_*p*_* = 0.145]. The results were also significant for the remaining retention levels but revealing a pupil constriction to “old” words compared with “new” words for lag 4 [*F* (3.123, 128.040) = 3.035; *p* = 0.030; η*^2^_*p*_* = 0.069], lag 8 [*F* (2.286, 93.725) = 4.169; *p* = 0.014; η*^2^_*p*_* = 0.092], and lag 32 [*F* (2.964, 121.504) = 3.343; *p* = 0.022; η*^2^_*p*_* = 0.075], as depicted in Figure 3.

### Pupil Dynamics to Recognition Errors

**FIGURE 4:**
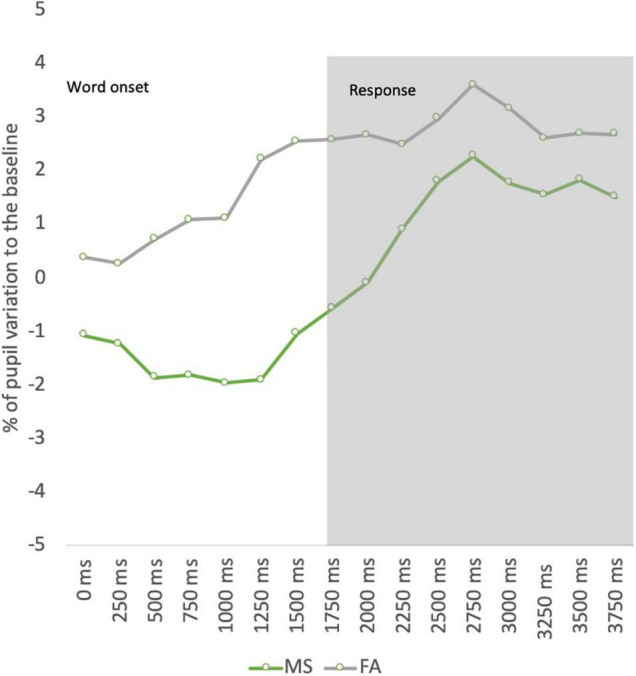
Pupil dynamics to recognition errors.

Pupil activity was also analyzed for recognition errors. According to the SDT, the failure in detecting an item presented previously at the learning phase is defined as a miss, whereas the failure to reject a new item (interference word) is defined as a false alarm. The comparison with the repeated measures two-factor (type of recognition error and bin) ANOVA revealed a significant main effect, suggesting an overall difference in pupil dilation between misses and false alarms, with increased pupil dilation for false alarms [*F* (1, 77.123) = 6.806; *p* = 0.023; η*^2^_*p*_* = 0.233]. No interaction effects were found indicating that the pattern of pupil activity is not different between the two types of recognition errors (Figure 4).

### Pupil Dynamics for False Alarms in High vs. Low Confident Decisions

**FIGURE 5:**
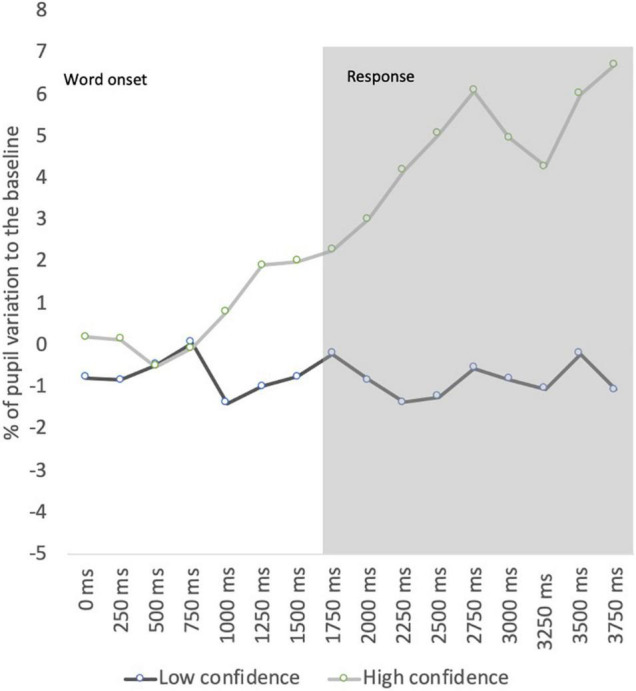
Pupil dynamics for false alarms in high vs. low confident decisions.

Given the increased response to false alarms, in which the mean percentage of pupil dilation to the baseline was 2.10%, being very similar to the mean dilation observed for words tested at lag 1 (2.49%), we have conducted a further analysis by confidence levels (low vs. high) for false alarms to analyze pupil activity in subjective familiarity decisions. The comparisons between high-confident responses (confidence rating of 5) and less-confident responses (confidence rating below 5) in false alarms show a marginally significant difference [*F* (1, 34.965) = 4.663; *p* = 0.054; η*^2^_*p*_* = 0.298] between the mean dilation to high-confident responses (2.9%) and less-confident responses (−0.78%), as depicted in Figure 5.

### Pupil Dynamics by Retention Interval for High-Confident Decisions

**FIGURE 6:**
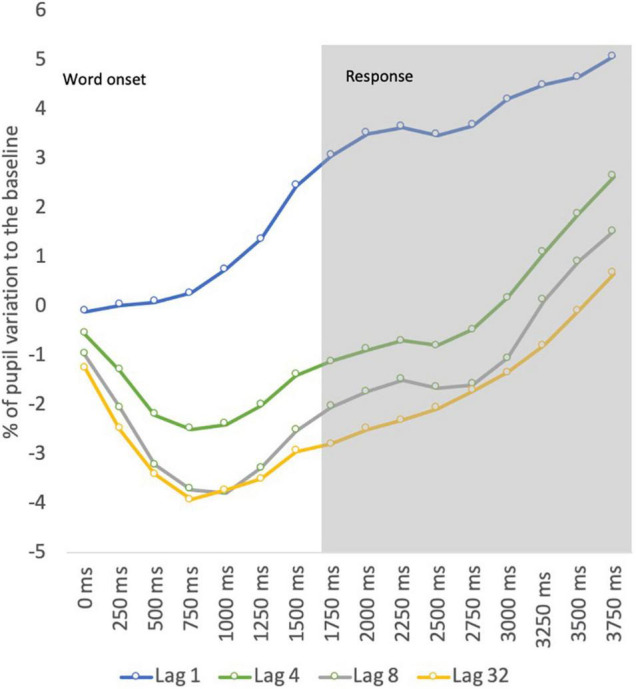
Pupil dynamics by retention interval for high confident decisions.

The above results suggest that pupil activity may be sensitive to subjective familiarity, which may occur when the participant rejects a “new” interference item probably being influenced by the subjective feeling of knowing that such an item was old. This may have been the case for extreme-confident decisions in false alarms. Therefore, the pupillary response by retention condition was reanalyzed only for extreme-confident decisions. The same two-factor ANOVA detected a main effect of retention condition [*F* (2.534, 101.376) = 20.328; *p* < 0.001; η*^2^_*p*_* = 0.337], showing the same temporal pattern across lag conditions. A main effect of bin was observed [*F* (3.068, 122.705) = 27.740; *p* < 0.001; η*^2^_*p*_* = 0.410], while the interaction effect [*F* (6.896, 275.846) = 4.868; *p* < 0.001; η*^2^_*p*_* = 0.108] revealed a decrease (Bonferroni corrected) in evoked pupillary responses across lag conditions providing similar results to that of the ANOVA without controlling for confidence ratings (Figure 6).

### Pupillary Response by Retention Interval According to the Number of Interference Items

**FIGURE 7:**
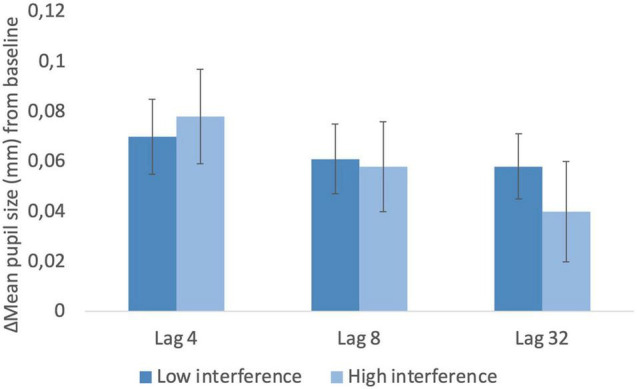
Pupillary response by retention interval according to the number of interference items.

In this experimental task, the intervening items separating study-test trials comprised both study words, “old” studied words and “new” test (interference) words. Interference varied according to the number of “new” test words separating the study-test trials. This variable related to interference was divided according to the median for trials with low interference vs. high interference. This analysis was conducted with a two-factor repeated-measures ANOVA (retention level with 3 levels: 4, 8, and 32 items and interference: low vs. high). The retention level 1 was not included as this condition consisted of immediate recognition. The ANOVA did not reveal significant effects of interference in pupil dilation (all *p*’s > 0.05), although the visual inspection to Figure 7 suggests an interaction between interference and lag condition on pupil activity.

## Discussion

This study aimed to investigate the relationship between pupil activity and recognition memory according to explicit manipulations of memory strength in a continuous recognition memory design. This goal was achieved by exploring pupil dynamics across different retention intervals to objective and subjective old/new status of word items in a running recognition task.

The behavioral data show a decrease in recognition performance with increasing retention intervals in recognition. The discrimination ability decreased with an increasing lag between study and test items, mostly in the transition from shorter retention (lag 1) to moderate retention (lag 4 and lag 8). Confidence ratings also decreased at longer retention levels, which distinguished shorter (lag 1), moderate (lag 4 and lag 8), and longer (lag 32) retention intervals. The results from reaction time were in the same direction but indicated an earlier impact in recognition performance from lag 1 to lag 4. Altogether, the behavioral results suggest that the recognition task was effective in manipulating memory strength as recognition performance decreased with an increasing lag between study and test of word items, but the increase in reaction times also indicates that effort may have increased across the retention intervals.

The pupil data revealed increased pupil dilation for words tested at lag 1. Likewise, the pupil old/new effect was found only for words tested at the shortest retention interval. The comparison between lag 1 and the remaining lag conditions showed that mean pupil dilation decreased as retention levels increased. These data contradict previous studies on working memory that suggest an increase in pupillary response when the number of items maintained into memory increased up to 4–5 items (Unsworth and Robison, 2018). Therefore, if the current results depicted working memory processing, we should expect an increase in pupillary response at least until lag 4 (i.e., four words between study and test), instead of the decrease observed from lag 1 to lag 4.

Our data also revealed that differences in the pattern of pupil activity across lag conditions were evident mainly by stronger pupil constrictions to items recognized at longer retention intervals. Considering that each study trial lasts approximately 2 s, the retention interval between the study and test phases for a stimulus tested at lag 4 is about 8 s, at lag 8 is about 16 s, and at lag 32 is about 64 s. Pupillary responses at lag 1 may correspond to a condition when the stimulus is still active in memory endorsing larger pupil dilations, comparing with longer retention levels when other memory processes may occur as an active rehearsal for long-term memory storage. This early constriction is not likely to be related to light reflex during the baseline period because in our study pupil baseline was calculated at 250 ms before the stimulus onset corresponding to a string of symbols to minimize the influence of luminance during the transition to the target stimulus while also preventing the accommodation effects on pupil size.

The study by van Rijn et al. (2012) also revealed an initial pupil constriction during word retrieval, but in our study, the size of this initial constriction seems to be associated with memory strength as this was more pronounced for items that were recognized at longer retention levels. In a previous study, using temporal analysis for pupillary response to complex stimuli (i.e., scenes) revealed that the initial constriction of pupil size during memory retrieval was related to novelty, where novel scenes elicited stronger pupil constrictions compared with familiar scenes in high confident decisions (Naber et al., 2013). In this study, this prediction was not possible to investigate as this would require novel items that were not familiar to the participants. In our study, we selected only high familiarity words to control for familiarity effects. A *post hoc* analysis to familiarity by splitting the data according to the median level of familiarity did not reveal significant effects on pupil data, although this result should be interpreted with caution given the low range of familiarity levels for item words used in this study, which varied from 1.1 to 3.5 for 4–7 letter words (Marques et al., 2007).

The decrease in pupil dilation across lag conditions contradicts the effort accounting that memory effort increases pupil dilation (e.g., Granholm and Steinhauer, 2004; van Rijn et al., 2012), as the increase in effort revealed by an increase in reaction times should have produced increased pupil dilations, but the reverse was found in our study. Another study found increased pupil dilation for study lists repeated once corresponding to a more effortful condition compared with items retrieved after more repetitions (van Rijn et al., 2012). One possible explanation for these differences may be related to the nature of the task employed in our study. In this running recognition task, performance at each retention interval may be affected not only by decay (time) but also by interference in an overall effect, which differs from tasks employing single lists of items that study words in isolation. The decrease found in pupil dilation across retention levels may be related to decay and interference as longer retention intervals imply more intervening items and longer periods of time between the study and test phases. The intervening items were words in a continuous sequence that comprised both study words, “old” studied words and “new” test or interference words, being the latter used to fill the sequence at each retention condition. To investigate whether interference through the number of interference words influenced pupil dilation, the test trials for each of the retention conditions were divided by the median number of interference items, which did not show significant effects on pupillary response. It is advisable that the future studies have to distinguish between the effects of decay (time) and the number of interference words in the recognition task. Moreover, the manipulation of repetition of test trials in an adapted version of this continuous recognition memory design will be crucial to study in more detail the effects of memory effort across lag conditions. The assessment of vigilance and fatigue levels will be also an important consideration for further studies. Despite this, recognition design may minimize the potential effects of fatigue, as the retention interval was randomly manipulated across the continuous recognition procedure, future studies should consider both *online* measures as eye blink analysis and *offline* self-reports for assessing fatigue levels in continuous recognition memory designs to better describe pupil activity.

Furthermore, the results were also explored regarding recognition errors. The data revealed that false alarms (new items judged as old) elicited an increased pupil dilation compared with misses (old items judged as new). These data are aligned with the results from Kafkas and Montaldi (2015) that found increased pupil dilations for false alarms compared with misses, which discriminated between an early component of pupil data reflecting the objective veridical status of old/new items and a late component reflecting the subjective status of old/new items. To explore whether the subjective recognition decision modulates pupillary response, our data were analyzed according to the confidence level in false alarms. The results indicate that pupils dilated more when participants believed a new item was previously seen during the sequence mainly for high-confident incorrect decisions. Nevertheless, the analysis of confidence effects in pupil size across the retention interval did not seem to modulate pupil response for correct decisions. This latter analysis may have been affected by the lack of sensitivity as most correct responses were accompanied by extreme-confident decisions. In fact, the ROC analysis shows that this variable did not discriminate recognition responses. Future studies should also use feasible confidence scales to distinguish confidence in recognition decisions more effectively.

In sum, these results point to a relationship between pupillary response with the strength of the underlying memory signal in light of the following data: (1) The increase in retention interval decreased overall pupil dilation; (2) the pupil old/new effect was evident only for the shortest retention level; and (3) the analysis on the dynamics of pupillary response revealed a different pattern of pupil activity across the retention interval. However, it is also important to note that this response may be dependent on the subjective feeling of familiarity to a given item, as pupil size was also modulated by incorrect recognition decisions to “new” interference words especially those with high confidence.

Given the simplicity and non-intrusiveness of a pupil size measurement, the development of reliable methods for assessing pupil activity may provide an ecologically valid measure for assessing human memory and behavior in complex environments. The integration of pupil size measurement in virtual reality environments need not wait for further research. For instance, Juvrud et al. (2018) have demonstrated that it is possible to have a method based on a virtual reality scenario for assessing pupillary responses not depending on low-level stimulus features. In such virtual reality environments, it will be interesting to explore the current results under naturalistic contexts using stimuli other than words (i.e., objects, faces) and test whether pupillary responses are associated with the strength of memory in conditions that resemble real-life situations. Likewise, the study of false memory in virtual reality environments will be also intriguing given the current results suggesting the sensitivity of pupillary response not only to the objective oldness of the items but also to the subjective feeling of familiarity that drives recognition decisions.

## Data Availability Statement

The raw data supporting the conclusions of this article will be made available by the authors, without undue reservation.

## Ethics Statement

The studies involving human participants were reviewed and approved by Comissão de Ética e Deontologia para a Investigação Científica – CEDIC. The patients/participants provided their written informed consent to participate in this study.

## Author Contributions

JO was responsible for designing the study and writing the initial version of the manuscript. MF conducted data collection and contributed to the initial version of the manuscript. PR was responsible for data processing procedures, whereas PG was involved in the statistical analyses. All authors have contributed and approved the final version of the manuscript.

## Conflict of Interest

The authors declare that the research was conducted in the absence of any commercial or financial relationships that could be construed as a potential conflict of interest.

## Publisher’s Note

All claims expressed in this article are solely those of the authors and do not necessarily represent those of their affiliated organizations, or those of the publisher, the editors and the reviewers. Any product that may be evaluated in this article, or claim that may be made by its manufacturer, is not guaranteed or endorsed by the publisher.
